# Food security at risk: the consequences of limiting animal source foods

**DOI:** 10.1093/af/vfae030

**Published:** 2025-04-05

**Authors:** Craig Gundersen, Lora Iannotti, Frederic Leroy

**Affiliations:** Hankamer School of Business, Baylor University, Waco, TX, USA; E3 Nutrition Lab, Brown School, Washington University, St. Louis, MO, USA; Industrial Microbiology and Food Biotechnology (IMDO), Faculty of Sciences and Bioengineering Sciences, Vrije Universiteit Brussel, Brussels, Belgium

**Keywords:** animal source foods, food insecurity, hunger, malnourishment, regulations, taxes

**Implications**

- Over three billion persons are food insecure across the world with almost one billion suffering from undernourishment, making this one of the leading challenges we face today. Ensuring access to high-quality foods through equitable food systems is central to alleviating food insecurity.
- The efficient functioning of the entire agricultural supply chain is critical to the promotion of economic growth and affordable foods. In recent years, there have been a series of policies and policy proposals that are targeted against animal source foods (ASF). This imposition impedes efficient functioning and leads to higher rates of food insecurity.
- Certain populations are highly vulnerable to malnutrition with restricted dietary intakes of ASF. These populations may be delineated by: 1) life course phase, including those with unique biological requirements (e.g., pregnant/lactating women, children and adolescents, and older adults); and 2) contextually, those with social, economic, or environmental barriers to accessing ASF.
- Anti-meat taxes and regulations are being imposed and proposed in high-income countries. One of the consequences of these interventions is higher rates of food insecurity due to higher prices. We estimate that up to three million more Americans will be made food insecure by the taxes alone, which amounts to almost a 10% increase in overall food insecurity rates.

## Introduction

Food security has been defined by FAO (1996) as the situation “when all people at all times have physical, social, and economic access to sufficient, safe, and nutritious food that meets their food preferences and dietary needs for an active and healthy life.” Today, over three billion persons in the world suffer from food insecurity, with almost a billion suffering from the more serious problem of undernourishment (FAO, 2023).

Food insecurity and its attendant consequences would likely be far worse were it not for the contributions of livestock agriculture to the production of animal source foods (ASF). In their role as safe, affordable, and nutritious foods that billions of people enjoy, they help to reduce food insecurity. Nonetheless, there are some actors in the food systems debate who are disproportionally critical of, if not overtly hostile to, animal husbandry and seek to sharply restrict access to meat, dairy, and other ASF by imposing regulations at the production level, limiting accessibility, or increasing prices (Leroy et al., 2023).

In this article, we provide context for the grave threat posed by such anti-livestock advocacy. In doing so, we are not dismissing arguments for reasonable transformation of the livestock sector (cf. Leroy et al., 2022), which will have to evolve given the current environmental and animal welfare challenges, but we are warning against the imposition of ill-conceived barriers that would imply lower global accessibility to ASF. Of particular concern is that these efforts would harm those who are most vulnerable, namely those who are food insecure or at-risk of being food insecure, including those with increased biological requirements for nutrients that are more bioavailable in ASF. While food insecurity and its consequences are far worse in low-income countries, the problem is also relevant for higher-income countries, where much of the actions against ASF in general, and meat in particular, are occurring.

## Policy Proposals to Restrict Access to ASFs

Various influential and vocal groups seek to forcefully restrict access to ASF. As a prominent example, the authors of the EAT-Lancet report have argued that “consumer whim” needs to be overruled (Willet et al., 2019), based on the expectation that consumers, given their own freedom to make food choices, would continue to eat meat. The tactics used to meet those goals consist of imposing regulations, fiscal interventions, taxes, and other impediments that will make it more difficult to produce and consume ASF. Several broad sets of proposals have been proposed by such groups as the CEAP/Guarini Centre (Minelli et al., 2021), World Resources Institute (Ranganathan et al., 2016), and WWF (Loken et al., 2020), ranging from “soft” (e.g., nudging, front-of-pack labeling, and nutritional guidelines) to “hard” policy interventions (e.g., meat taxes or bans). Various scientists have proposed specific techniques to be employed to this end, including the use of “compassion inducing” restaurant decor, the discoloration of meat to make it unappealing, meat shaming and broader anti-meat communication campaigns, as well as the outright banning of meat from restaurant menus (for references and other examples of interventionism, see Leroy et al., 2023).

In addition to measures that target consumer behavior, impediments at the other end of the supply chain, in the production of ASF, have also been considered. A longstanding method by “animal rights”-inspired groups is to disrupt production either through direct actions or legislation. For example, legislation has passed in California and Massachusetts that dictates the housing of chickens and hogs. A more recent set of proposals is to force production changes to be more “environmentally friendly,” disproportionally targeting livestock. While we are not dismissing the fact that the livestock sector needs to become more sustainable, the more radical proposals are typically based on a misrepresentation of the interaction between animal agriculture and the environment (Manzano et al., 2023).

Taxes on ASF have often been proposed as a tool to curb demand, especially for meat and, to a lesser degree, for dairy (Caillavet et al., 2016; Bonnett et al., 2018; Springmann et al., 2018; Funke et al., 2022). The level of the taxes differs depending on the goals stated (e.g., reducing a particular negative health outcome by a certain amount), the type of food considered (e.g., reducing beef consumption to obtain a particular level of greenhouse gas emissions), and the region, i.e., low-income countries (LICs) vs. high-income countries (HICs). Such proposed tax increases (usually 20% or more) would be considerably higher than any of the existing sales taxes on food in the United States, where only some states tax food and even then, generally no more than 5% with the exemption of purchases made through the Supplemental Nutrition Assistance Program (SNAP, formerly known as the Food Stamp Program).

## Global Food Security

Across the world, over three billion people are food insecure with 25% of those suffering from undernourishment (FAO, 2023). This masks immense disparities in food insecurity, with Africa and Asia regions disproportionately affected. Over half of preschool-aged children (56%) and two-thirds of nonpregnant women of reproductive age (69%) have at least one of three micronutrient deficiencies in iron, zinc, and/or vitamin A (Beal and Ortenzi, 2022). The agrifood framework encompasses different aspects of agriculture and food systems that can lead to food insecurity (Iannotti et al., 2024). Specifically, food environment elements include availability, affordability and access, promotion, quality, and sustainability (Downs et al., 2020).

Contemporary drivers of food insecurity worldwide are poverty, environmental challenges and extreme weather events, armed conflict, and political instability. Exacerbated by the COVID-19 pandemic, significant increases in food insecurity arose in some countries with food price inflation and compromised livelihoods. Other shocks including Russia’s war in Ukraine and ongoing armed conflicts in Israel, Haiti, Yemen, Somalia, Sudan, Afghanistan, and Central African Republic have forced millions more into hunger. Extreme weather events (floods, tropical storms, and cyclones) or sustained drought have worsened conditions of moderate and severe food insecurity, largely captured empirically through food production pathways (Dasgupta et al., 2022; Hadley et al., 2023). Here we focus on policy and regulatory constraints in the livestock sector that may differentially affect small- and medium-scale producers, already vulnerable to the impacts of climate change, poverty, and malnutrition (Herrero et al., 2017).

We wish to especially emphasize the role of poverty as an impediment to food security in LICs. The ability of a country’s residents to be food secure depends, in part, on a country’s overall wealth. With respect to broad historical patterns, in 1820, 94% of the world’s population lived on less than $2 per day making securing enough food throughout the year a serious challenge. By 2015 this had fallen to less than 10% (for more on the data used for these calculations, see Ravallion, 2016). This dramatic drop in extreme poverty is ascribed to the burgeoning of economies across the world, albeit with larger growth in some areas than others (McCloskey, 2019). As extreme poverty falls, so too does the extent of food insecurity.

## Public Health Consequences of Interventionism Targeting the Livestock Sector

Restricting access to ASF can have dire food insecurity and, more broadly, health consequences, especially for vulnerable populations, which are largely neglected in both the evidence base and policy discourses. These populations may be delineated by periods in the life course and by context—social, economic, and environmental conditions. Epidemiological evidence supporting plant-based diets centers around adult populations living in HICs where purchases of many types of foods are feasible (Oussalah et al., 2020). However, in food-insecure populations, and for those in life periods with special nutrient needs, restricting access to ASF can precipitate poor health. Here, we highlight those vulnerable populations recognized by dietary guidelines worldwide to regularly require ASF.

### ASFs in the life course

Over thousands of years, humans have evolved to require different nutrients in the diet to support growth, development, reproduction, and maintenance of health across the life course. One defining characteristic of *Homo sapiens* relative to other primates and some animals more broadly is the ability to adapt to multiple environments and dietary patterns. Dietary diversity has been the hallmark of many foraging hominid species until only recent time with the emergence of agriculture and nation-state boundaries (Eaton et al., 2017; Iannotti et al., 2022). Animal foods were integral to these patterns and have been associated with such physical characteristics in hominids as taller stature and larger brain size (Kuipers et al., 2012; Leroy et al., 2023). Here we discuss the contemporary biological needs for ASF emerging from our long evolutionary past, highlighting those phases in the life course when restriction can lead to poor health outcomes.

Evidence from experimental or quasi-experimental trials generally tests the addition of ASF to usual diets (instead of withholding or restricting) on health outcomes. By contrast, observational studies are more often used to study dietary patterns such as vegetarian, lacto-, pesco- and/or ovo-vegetarian, and draw inferences regarding health consequences, most commonly in adults. One recent umbrella review of 20 meta-analyses found that vegetarian diets, inclusive of all types, compared to omnivore dietary patterns were associated with lower blood cholesterol, but showed harmful outcomes on one-carbon metabolism markers such as vitamin B12 and homocysteine levels (Oussalah et al., 2020). Here, we cover the health advantages of ensuring consumption of ASF in vulnerable periods of the life course.

Nutritional requirements vary across the life course to sustain biological systems and varying processes. Certain periods such as the first 1,000 days of life necessitate ASF—as foods with highly bioavailable nutrients enabling efficient absorption and metabolism. Pregnant and lactating women require high levels of protein, iron, calcium, and choline particularly, but an even broader range of nutrients is needed to support blood plasma expansion, fetal growth and development, milk production, and multiple other processes (Allen, 2013). Evidence on ASF during pregnancy and lactation strongly indicates that milk and dairy products are beneficial for birth outcomes and newborn anthropometry (Achón et al., 2019; Pérez-Roncero et al., 2020). Complementary feeding from 6 to 24 m is another critical phase for ASF (Eaton et al., 2019; Shapiro et al., 2019). The WHO has now included recommendations for ASF as part of dietary diversity in their recent complementary feeding guidelines (WHO, 2023).

During middle childhood and adolescence, ASF are an important part of diets to support ongoing brain development, puberty, growth, and other rapid changes in this period. Rapid neurogenesis and synapsis formation unfolds, followed by a period of intense pruning during adolescence (Black et al., 2017). Docosahexaenoic acid (DHA), iron, zinc, choline, and B vitamins found in ASF are all crucial for brain development. Evidence again supports the inclusion of milk and dairy products for this age group (de Beer, 2012; Lu et al., 2016). A study conducted in Kenya showed the importance of meat and milk in school-age children for cognition, anthropometry, and morbidities (Neumann et al., 2007, 2012, 2013). Older adults have special dietary needs to sustain physiological processes ongoing in aging—senescence of replicating cells, oxidative stress, muscle wasting, bone loss, and cognitive declines (Solomons, 2013). There is consistent and strong evidence, primarily from HICs, suggesting red meat consumption has positive impacts on muscle health (Rondanelli et al., 2015; Granic et al., 2020), while milk and dairy can mitigate sarcopenia (muscle loss), fractures, frailty, dementia, and Alzheimer’s disease (Cardoso et al., 2016; Cuesta-Triana et al., 2019; Bermejo-Pareja et al., 2021).

### Contextual factors and access to animal source foods

Vast inequities exist for accessing ASF. Notably, resource-poor contexts have limited access to ASF and will be especially vulnerable to malnutrition with further restrictions. Access ASF varies greatly by region—as indicated by ASF supply as a percent of total calories for Western high-income regions (around 30%), Central and South America (around 20% to 25%), most of Asia (around 10% to 20%), and most of Africa (around 5% to 10%; Leroy et al., 2024). Nationally representative data reveals income-driven disparities for young child consumption of ASF, with dire consequences as described above. While low generally, the percentage of children aged 6 to 23 m from low- and middle-income countries (LMIC) reported receiving any ASF varies depending on wealth quintile, from poorest to wealthiest: dairy 36% to 64%; flesh foods including meat, fish, poultry, and organs 25% to 39%; and eggs 17% to 30% (UNICEF, 2021). In low-resource settings, there is a heavy reliance on affordable staple foods such as corn, rice, and cassava leading to low diet diversity and malnutrition (Reeves et al., 2016).

**Figure 1.**
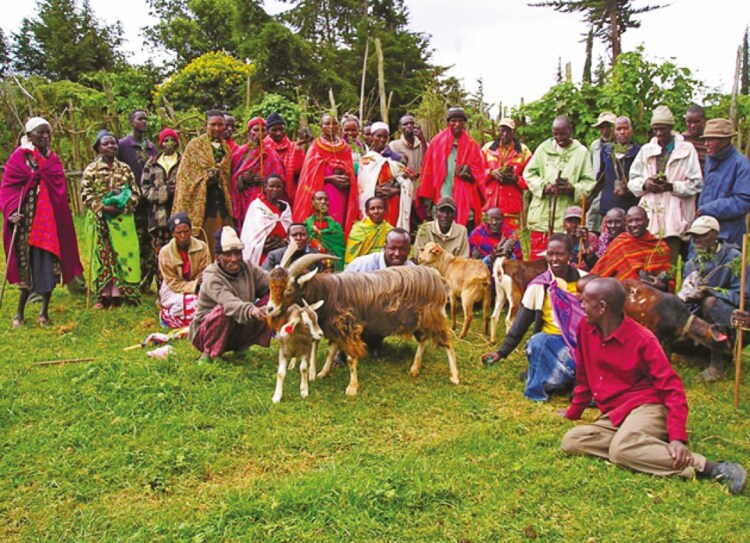
Samburu pastoralists (photo credit: C. Lesorogol).

Beyond economic drivers, there are social, political, and environmental factors that can diminish access to ASF with public health consequences. Humanitarian crises and natural disasters can disrupt ASF supply chains and block access to ASF. While this may occur on a short-term basis, individuals in a sensitive life course phase such as the first 1,000 days may endure morbidities or mortality. Malnutrition in this period can have life-long consequences jeopardizing brain development, physical growth, and reproductive health (Black et al., 2013). Food aid donations can ensure food security in these crises, but tend to be comprised of staple commodities produced in HICs including corn, soybean, and rice, with low nutritional value and rarely including ASF (Webb et al., 2017). Severe weather events such as floods or drought harm agrifood systems and reduce livestock production among small holders leading to food insecurity and reduced access to ASF (Thornton et al., 2009; Dzavo et al., 2019). Small-scale producers of ASF (livestock, fisheries, aquaculture) in LMIC have limited access to inputs such as loans, extension services, and subsidies, reducing efficiencies and overall production. Pastoralists or indigenous communities may be further marginalized socially and economically leading to reduced ASF access and food insecurity (Box 1, Figure 1).

Box 1. **Pastoralists**

Pastoralists are among the most vulnerable populations in the world today. Between 200 and 500 million persons are pastoralists with approximately one billion animals under their care including camels, cattle, goats, horses, reindeer, sheep, and yaks. Pastoralist systems are crucial for food security in the Sahel region of Africa but also globally in areas where the cultivation of crops presents challenges. Further, pastoralist systems can support sustainable rangelands through increased soil fertility, nutrient cycling, and biodiversity conservation (FAO, 2024).

For thousands of years, pastoralists have depended on ASF for their own nutrition. In recent decades, however, climate change, conflict, and diminishing land tenure have converged to force pastoralists to diversify livelihoods and into greater food insecurity. A longitudinal study of the Samburu pastoralists in Kenya was conducted in which it was found that milk, once integral to their diets and critically important for providing a range of nutrients, now only comprises 10% of calories, well below that of maize (52%; Iannotti and Lesorogol, 2014a). Undernutrition was highly prevalent among these Samburu children reflected in high rates of stunting (30.6%), underweight (23.9%), and wasting (8.6%). Milk consumption was found to be protective of BMI, while livestock ownership predicted nutrient adequacy for vitamin A, B12, and zinc (Iannotti and Lesorogol, 2014b). It is vitally important that policies and programs support pastoralist livelihoods and ASF in their diets to ensure food security and well-being.

## Food Insecurity in HICs

As discussed above, context matters enormously for access to ASF and the resulting health consequences. The extent, magnitude, and consequences associated with food insecurity in LICs far exceed those found in HICs like the United States. For example, stunting and wasting occur in LICs, these are rare in HICs. Nevertheless, we cover food insecurity in HICs, with an emphasis on the United States, insofar as many of the proposals to restrict access to ASF covered above are directed, especially in the near term, to HICs. We concentrate on three determinants that are especially influenced by efforts to curtail access to ASF, namely economic growth, food prices, and SNAP (for a broader discussion of the determinants of food insecurity, cf Gundersen and Ziliak, 2018).

### Economic growth

**Figure 2.**
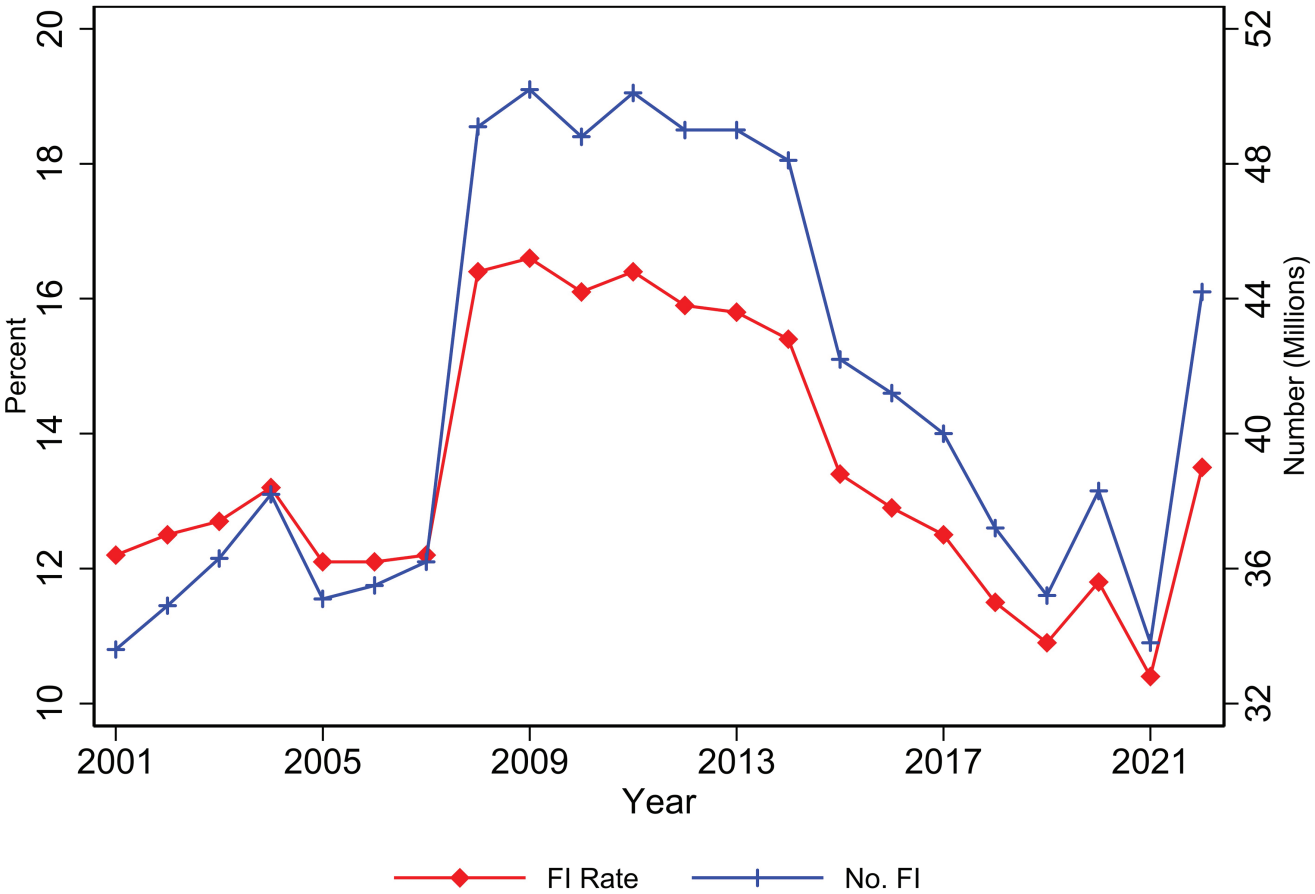
Trends in food insecurity, full population, 2000 to 2022.

As discussed above, long-term economic growth is responsible for dramatic declines in food insecurity across the world. A similar story holds in both the short and long run for food insecurity in the United States. Concentrating on the short run, as seen in Figure 2, the highest food insecurity rate was in 2009 during the peak of the Great Recession. By 2021, rates had fallen by 37%. For children, the decline was even larger, namely 45%. Over this period, measures of underlying economic conditions also improved. As examples, nominal Gross Domestic Product rose by 72% (https://fred.stlouisfed.org/series/GDP) and real median household income rose from $65,850 to $76,330 (Guzman and Kollar, 2023). In addition to overall economic growth, Americans moved to areas with higher economic growth and, hence, were more likely to escape food insecurity. This can be seen in a comparison of what has happened in the Midwest and the South due to the large migration to the latter in response to higher economic growth. In 2010, the food insecurity rate in the South was 2.6 percentage points higher than in the Midwest (17.6% and 15.3%). By 2021, the rates were only 0.5 percentage points apart (11.5% and 11.1%). For children, a similar narrowing holds and rates actually became lower in the South (14.8% and 15.0%; Gundersen, 2023).

Economic growth in the United States and elsewhere relies on industries producing jobs, expanding operations, and creating products at competitive prices. In the United States alone, the meat and poultry industry produced 28 billion pounds of beef, 28 billion pounds of pork (USDA and NASS, 2022a), and 50 billion pounds of poultry (USDA and NASS, 2022b) with a total value of almost $230 billion (https://data.census.gov/table?q=31-33:+Manufacturing&tid=ASMAREA2017.AM1831BASIC02&hidePreview=false). The meat and poultry industry alone employs over a half-million persons (https://www.census.gov/data/tables/2019/econ/susb/2019-susb-annual.html). These figures do not include all of the further billions of dollars produced and millions of people employed at other stages of the agricultural supply chain. Along with being an extraordinarily large industry in the United States, the meat industry has made continuous improvements to the production process which leads to an ever-more efficient industry. As an example, over the last six decades, pork production has almost doubled (Putnam et al., 2018). Despite this large increase in production, the amount of resources devoted to pork production has declined dramatically; 75% less land, 25% less water, and 8% less energy are being used (We Care, 2021). The efforts to regulate and tax the meat industry will do great damage to this engine of economic growth. This decline in economic growth will then lead to higher food insecurity.

### Food prices

For many Americans, the proportion of disposable income spent on food is relatively small (10% or less), but for lower-income Americans, this can be far higher (up to 20% or more). Higher food prices, then, have a larger impact on low-income households. As it pertains to food insecurity, research has shown that higher prices do lead to higher levels of food insecurity and nutrient inadequacies (Courtemanche et al., 2019; Zheng et al., 2021). The same holds for LICs (Iannotti and Robles, 2011; Iannotti et al., 2012, 2022).

The impact of higher food prices was seen in 2022. From 2021 to 2022, food insecurity rates rose from 10.2% to 12.8%, a 26% increase. This was the largest increase since the Great Recession and food insecurity rates reached their highest levels since 2014. While some of this increase is due to changes in the structure of the survey used to garner this data (the Food Security Supplement in the Current Population Survey; CPS), the rest of the increase can be ascribed to food price inflation which rose to 9.9% in 2022 (Rachidi and Gundersen, 2024). This should be contrasted with the average food price inflation of 1.7% from 2010 to 2019.

The imposition of taxes leads to higher prices and, therefore, when taxes are implemented this leads to higher rates of food insecurity. As it pertains to taxes on meat, this will lead consumers to choose suboptimal bundles of food. Given the change in the relative prices of meat, this then means they will consume less meat (the substitution effect) and less of other food items (the income effect). This will leave them worse off and more likely to be food insecure. How this translates into a price increase overall with respect to food depends on the portion of meat in people’s diets, the relative prices of other products, and other factors.

Caillavet et al. (2016) have quantified the impacts of food price increases. They examined the case whereby taxes on meat were increased by 20% and found that this leads to declines in food budgets by about 4% to 8%. Based on this decline in food budgets, the projected changes in food insecurity can be estimated. Along with the questions needed to calculate food insecurity on the CPS, a question is posed about the usual expenditures on food per week for a household. From this question, we calculate the average food expenditure per person per meal. With data from the 2022 CPS (the most recent year available), we regress food insecurity on usual food expenditures per meal. Doing so, we find that a 1% decline in usual food expenditures would lead to 60,000 more food-insecure households in the United States.

We use the results from this regression to calculate the impacts of the proposed increases in taxes on meat discussed above, as outlined in Table 1. In the first column of the table, the articles and the range of taxes proposed are displayed. In the second and third columns, then, we display the impact of these taxes on food insecurity based on the bounds established by Caillavet et al. (2016). If there is a 20% increase in taxes on meat, they estimate that there will be a decline of 4% to 8%; the impacts are displayed in the first row. As seen there, a 20% tax on meat leads to between 257,000 and 519,000 more food-insecure persons in food-insecure households. The other rows work under the assumption that there is a proportionate decline in lower or higher taxes. For example, a tax of 10% on meat would lead to 2% to 4% declines in food expenditures while a tax of 40% would lead to 8% to 16% declines in food expenditures. The largest impact of currently proposed taxes would be if a 111% tax on processed beef was implemented; this would lead to between 1.5 and 2.9 million more food-insecure persons in the United States. This is an upper-bound estimate insofar as this is only being proposed for processed beef products. Even so, the price of substitute goods (e.g., other meat products) increases when there is a tax on one good meaning while this is an upper-bound, the increase in food insecurity will be substantial when the increase in the prices of other goods and the subsequent substitution and income effects are calculated. The results in Table 1 demonstrate the profound consequences of potential meat taxes in the United States.

**Table 1.** Simulated impact of proposed meat taxes on the number of food-insecure persons (millions) in the United States

	Lower-bound estimate	Higher-bound estimate
Caillavet et al. (2016)		
20% tax	0.256	0.520
Springmann et al. (2018)		
21% tax	0.270	0.546
111% tax	1.452	2.908
Funke et al. (2022)		
20% tax	0.256	0.520
60% tax	0.782	1.570
Bonnet et al. (2018)		
12% tax	0.152	0.310
41% tax	0.533	1.071

Simulations based on data taken from the 2022 Current Population Survey (CPS).

The impacts of regulation on meat on food insecurity are difficult to identify but discussions of losses in consumer welfare—which is tied to food insecurity—have been established. While not a meat product, an illustrative example in the United States, is the recent move in some jurisdictions to only allow the sale of eggs from “cage-free” chickens. Oh and Vukina (2022) examine the impact of legislation in California on consumer welfare. They find that the implementation of this would lead, in California alone, to losses of $72 million per year. A regulation directly applied to meat is Proposition 12 in California which dictates the confinement size for hogs. This policy leads to a 20% increase in the prices of pork products in California (Hawkins et al., 2024), which results in a loss to consumers of $300 million (Sumner, 2024). Since California is such a large market and producer, spillovers to other states will lead to welfare losses across the United States.

### Supplemental Nutrition Assistance Program

The central tool used to reduce food insecurity in the United States is SNAP. Recipients obtain benefits through an electronic benefit transfer card that can be used to purchase food at virtually all retail food outlets. This is by far the largest program run by the U.S. Department of Agriculture, and its size can be seen in Figure 2, which shows the number of people enrolled and total expenditures on SNAP from 1980 to 2022. The number of participants roughly doubled from 1980 to 2023, with a peak of 47 million recipients in 2013. The number of SNAP recipients and expenditures increase during economic downturns (e.g., in 1990) but in recent years, both have remained high even after recessions end. COVID did have a slight impact on the number of recipients which rose from 36 to 42 million from 2019 to 2023. The number of recipients in 2023 was still less than in 2017. The impact on expenditures were substantially larger—from $56 billion to $107 billion. This is primarily because of a temporary increase in all SNAP benefits during COVID-19 and the permanent increase due to a 20% increase in benefits in 2021. An extensive literature has demonstrated the success of SNAP at reducing food insecurity (for a review, see Smith and Gregory, 2023).

**Figure 3.**
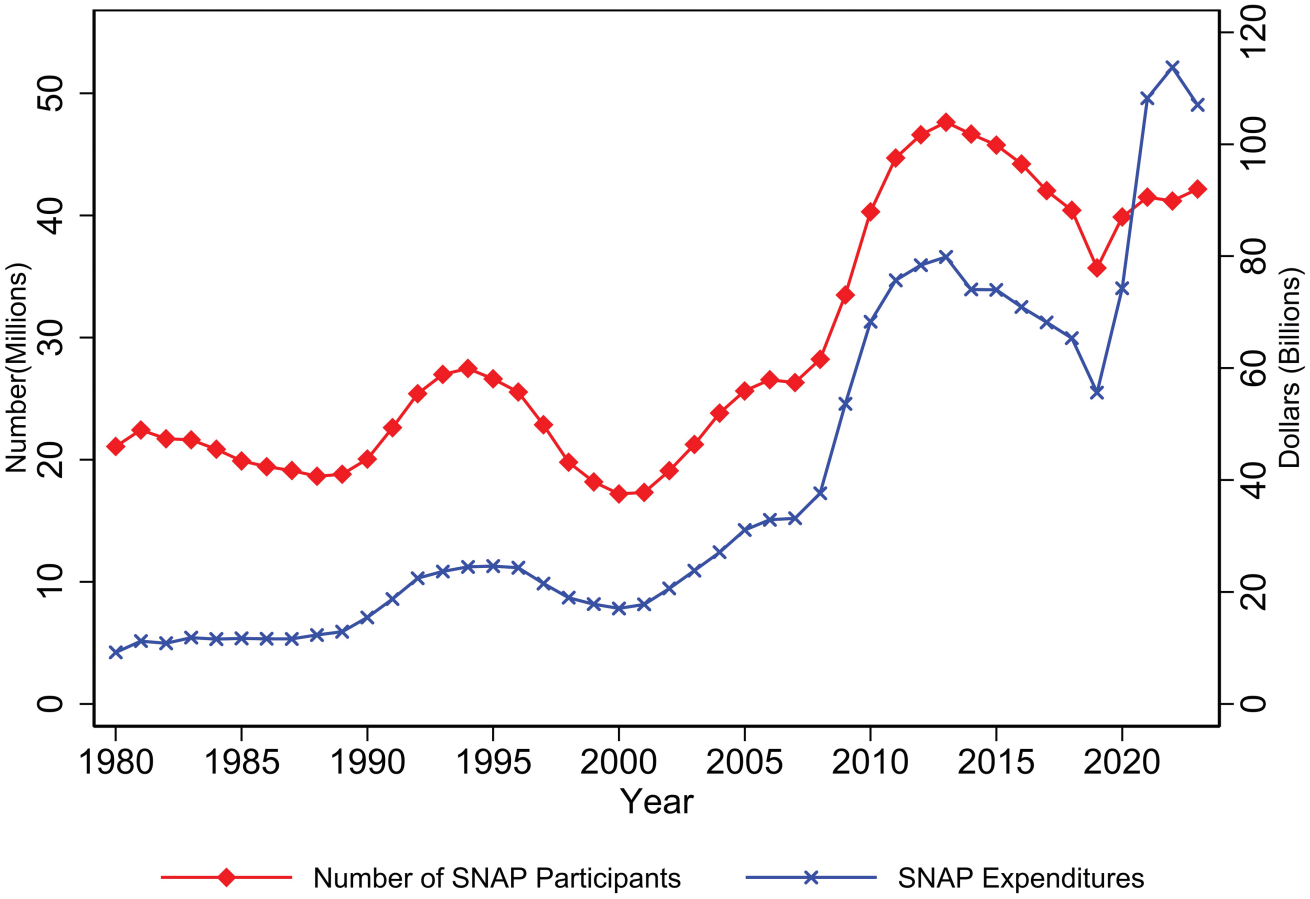
SNAP participants and expenditures, 1980 to 2023.

The success of SNAP in improving the well-being of vulnerable Americans and, in particular, by reducing food insecurity, can be attributed to four main factors. First, the program reaches those most in need. To be eligible for SNAP, a household must have gross income below 130% of the poverty line (approximately $30,000 for a family of four), net income below the poverty line, and assets below roughly $3,000 (many states waive the asset test and/or set a higher gross income threshold). Second, SNAP leverages the traditional retail sector rather than having, say, a separate set of locations to obtain food. It does so by allowing SNAP to be used at the almost 260,000 stores that accept SNAP beneﬁts (https://www.fns.usda.gov/data/snap-retailer-management-dashboard). Third, SNAP is an entitlement program. It has this stature insofar as, as seen in Figure 3, SNAP expands or contracts over time based on the need for benefits, primarily driven by economic conditions. This occurs without any explicit need for policymakers to fund additional expenses needed for the program. This differs from other programs where funding is capped. It is also an entitlement program whereby, with a few exceptions, persons can be on the program for as desired just so long as eligibility is maintained. Fourth, unlike some food assistance programs and other assistance programs in the United States, SNAP does not take away the dignity and autonomy of recipients (Gundersen, 2020). Recipients have dignity insofar as they are allowed to shop at the same stores and the same manner as their family, friends, and neighbors. They are also given the autonomy to make their own decisions about food that are consistent with their preferences, religious beliefs, dietary requirements, etc. This respect for the dignity and autonomy of recipients is one of the reasons for such high participation rates among eligible households, especially those with children.

Given its size and success, SNAP has made itself into a target for those who wish to use government interventions to change food consumption patterns. This has manifested itself in various calls to prevent SNAP benefits from being used to purchase items like sugar-sweetened beverages, candy, processed foods, and meat. In particular, there have been calls by some—including many who are also pushing for more regulations on meat and higher taxes on meat products—to only allow SNAP benefits to be used for purchases of food allowed by the Special SNAP for Women, Infants, and Children (WIC). The only ASF that is allowed to be purchased with WIC benefits is small quantities of canned tuna fish. Consequently, virtually all meat products would no longer be allowed in SNAP if the calls to have the WIC rules applied to SNAP are implemented. Removing meat from allowable purchases by SNAP recipients would have grave implications.

For all consumers, this would have negative implications as stores would be required to invest in and maintain an extensive set of technological and other interventions to flag SNAP ineligible products under these proposed restrictions. This would be a daunting task given that there are over 600,000 food products with UPCs in the United States and about 15,000 are added each year. Food stores would need to make decisions about which of these products would be on their shelves, with the average food store having about 40,000 products with associated UPCs. And the costs would repeat themselves, as they would be incurred when the restrictions are first imposed, every time a new product is introduced, every time a product switches a UPC code for other reasons, and so on. In addition to matching the UPC code to a list of approved food items, stores would have to decide how best to label if foods are eligible for SNAP. The above costs are those that would directly increase food prices. There would also be indirect costs, including longer checkout lines due to the complexity of ascertaining which products are and are not eligible for SNAP. As discussed in the previous sub-section, these increased costs would lead to increases in food insecurity.

In addition to higher food insecurity rates due to higher food prices, there would be declines in SNAP participation due to restrictions that would also raise food insecurity rates. As mentioned above, one of the strengths of SNAP is giving recipients the dignity to shop for the same products as other consumers and the autonomy to make those decisions. When those things are taken away, participation falls. One example of a program that does not give dignity and autonomy to recipients is WIC. Not surprisingly, participation rates among non-infants are very low. For example, despite much more lenient eligibility criteria, participation rates among four-year-old children in low-income households are half those found in SNAP. Given the critical role SNAP plays in reducing food insecurity, falling participation rates would lead to rising food insecurity rates.

## Conclusion

Hard policy measures targeting the animal agricultural industry and driven by anti-livestock sentiment generally operate under the guise of things such as “improving nutritional outcomes” and “addressing environmental concerns.” Strategies consist of restricting production through regulations and other means, including the use of food taxes. These actions will have grave consequences for the livelihoods of tens of millions across the world with direct connections to animal agriculture. (See Tonsor (2018) for a broader discussion of challenges in meeting these policy measures for agricultural producers.) What has been less publicized are the implications of these regulations and taxes on the well-being of billions of vulnerable persons across the world. In particular, what has been overlooked is the increase in food insecurity and associated negative outcomes. Any thorough consideration of the costs associated with actions geared towards harming the animal agriculture industry must look beyond the direct costs to that industry and also consider the costs for those most at-risk of food insecurity across the world.

## About the Authors

 **Craig Gundersen** Craig Gundersen is the Snee Family Endowed Chair at the Baylor Collaborative on Hunger and Poverty (BCHP) and a Professor in the Department of Economics at Baylor University. He is also the lead researcher on Feeding America’s Map the Meal Gap project, a Round Table Fellow of the Farm Foundation, and a Faculty Affiliate of the Wilson Sheehan Lab for Economic Opportunities (LEO) at the University of Notre Dame. His research concentrates on the causes and consequences of food insecurity and on the evaluation of food assistance programs, with an emphasis on SNAP. Gundersen is a Fellow of the Agricultural and Applied Economic Association (AAEA). **Corresponding author**: craig_gundersen@baylor.edu

 **Lora Iannotti** is a professor of public health and founding director of the E3 Nutrition Lab at Washington University in St. Louis. Her lab aims to identify nutrition solutions that embrace principles embodied in the three E’s: equity, environment, and evolution. Dr. Iannotti leads projects in Ecuador, Haiti, and Madagascar where she collaborates with local partners to test innovative approaches to achieving sustainable, healthy dietary patterns that improve young child growth and brain development. Her research related to animal source foods has informed the global discourse on nutrition equity, climate change, and planetary boundaries. Dr. Iannotti is Director of Planetary Health for the newly launched Center for the Environment at Washington University in St. Louis. Iannotti received her doctorate from the Johns Hopkins University Bloomberg School of Public Health, and a Master of Arts Degree in Foreign Affairs from the University of Virginia.

 **Leroy Frédéric** graduated as a Bioengineer (Ghent University, 1998) and obtained a PhD in Applied Biological Sciences at the Vrije Universiteit Brussel (VUB, 2002), where he now holds a professorship in food science and (bio)technology. He is primarily affiliated with the research group of Industrial Microbiology and Food Biotechnology (IMDO) and, additionally, with the research group of Interdisciplinary Historical Food Studies (FOST). His research deals with animal source foods, food processing and technology, food fermentation and microbiology, human nutrition, food systems and sustainability, and interdisciplinary “food studies.”
